# Supplementary material for: Genomic and Transcriptomic Analysis for Identification of Genes and Interlinked Pathways Mediating Artemisinin Resistance in Leishmania donovani
Source: Genes (Basel). 2020 Nov 17;11(11):1362. doi: 10.3390/genes11111362 (PMC7698566; doi:10.3390/genes11111362)
Supplement: Supplementary file 1 [file genes-11-01362-s001.pdf]

**Supplementary Information**

**Genomice and Transcriptomic Analysis for Identification of Genes and Interlinked Pathways Mediating Artemisinin Resistance in *Leishmania donovani***

Sushmita Ghosh[1,2], Aditya Verma[1], Vinay Kumar[1], Dibyabhaba Pradhan[3], Angamuthu Selvapandiyan[2], Poonam Salotra[1], Ruchi Singh[1*]

1. ICMR- National Institute of Pathology, Safdarjung Hospital Campus, New Delhi-110029, India

2. Jamia Hamdard University-Institute of Molecular Medicine, New Delhi-110062, India

3. ICMR-AIIMS Computational Genomics Centre, Indian Council of Medical Research, New Delhi- 110029

*Correspondence: ruchisp@gmail.com

## Supplementary Figures and Tables:

### Figure S1.

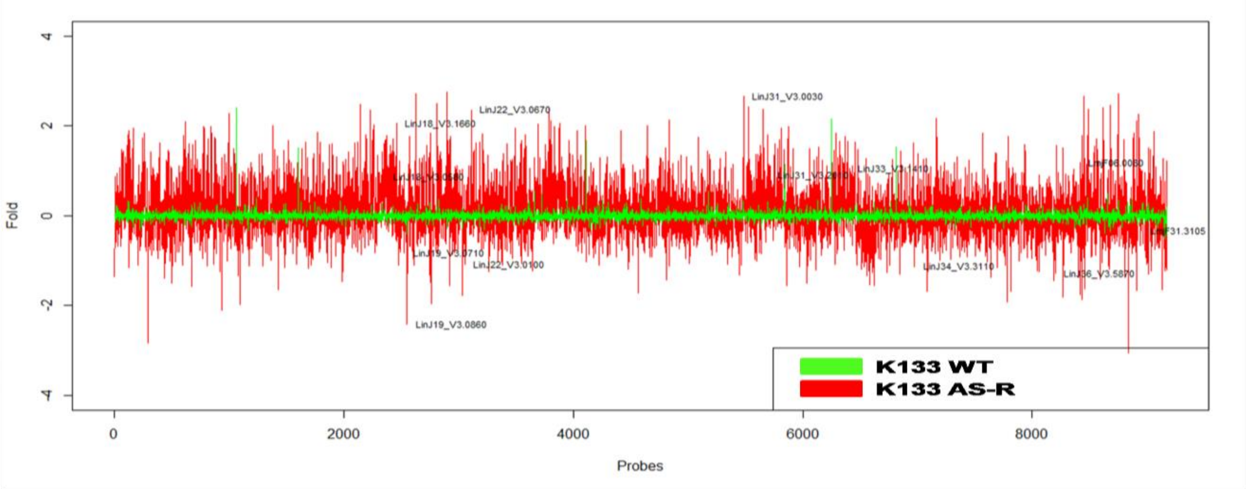


**Figure S1: Comparative transcriptional responses following ART adaptation in *L. donovani*.** Overlap of log2 transformed K133 AS-R and K133 WT expression ratio plotted as a function of chromosomal location of probes representing the full genome microarray. The plot represents the average values of three independent hybridizations for each isolate.

**Table S1: List of genes validated for their modulated expression by Quantitative real time-PCR**

| S.No. | Primer Name | Gene Name/ Gene ID | Function/relevance | Primer Sequence |
|---|---|---|---|---|
| 1 | AQP1 | Aquaglyceroporin (LinJ.31.0030) | Metal ion transmembrane transporter activity, integral to membrane; transmembrane transport; transporter activity; water transport. | F-5′CAGGGACAGCTCGAGGGTAAAA3′<br><br>R-5′GTTACCGGCGTGAAAGACAGTG3′ |

| | | | | |
|---|---|---|---|---|
| 2 | A2 | A2 protein (LinJ.22.0670) | Cellular response to stress | F-5′GTTGGCCCGCTTTCTGTTGG3′<br><br>R-5′ACCAACGTCAACAGAGAGAGGG3′ |
| 3 | ABCG1 | ATP-binding cassette protein subfamily G, member 1 (LmjF.06.0080) | ATP binding, ATPase activity, coupled to transmembrane movement of substances, phosphopantetheine binding | F-5′GATCTTCTTGAATGTGCGGGCC3′<br><br>R-5′CCACCAGCACTGCAAGAATCTG3′ |
| 4 | GATase | Glutamine aminotransferase (LinJ.33.1410) | Putative cysteine conjugate beta-lyase,aminotransferase - like proteinbiosynthetic process | F-5′GCAGGCTCTCAACGCCATATTC3′<br><br>R-5′AGCTTCCAGTTCTCAGCGGATT3′ |
| 5 | PECH | peroxisomalenoyl-coahydratase (LinJ.18.0580) | Putativeenoyl-CoA hydratase activity, Fatty acid metabolism | F-5′AGATTATCCAGCGCTCCTCACC3′<br><br>R- 5′ TTGATGGCAGCTTGGTACTCGA 3′ |
| 6 | ATPP | autophagocytosis protein (LinJ.33.0320) | autophagy; cytoplasm; protein transport | F-5′CCCTGAGAGCGTGCAAGACT 3′<br><br>R-5′CGCCAGAGTCATCGTCGTCT 3′ |
| 7 | Tpx | Tryparedoxin-like protein (LinJ.31.2010) | Hypothetical protein, involved in redox metabolism in trypanosomatids | F-5′CTCATCTGCTTCTCCGCCCA 3′<br><br>R-5′CAGTCTCGGCCGATCGTCTG3 |

| | | | | |
|---|---|---|---|---|
| | | | | ′ |
| 8 | VPS | vacuolar protein sorting-like protein (LinJ.25.2280) | vesicle docking involved in exocytosis vesicle-mediated transport | F-5′GTTCTTAGCAGGGTGTACGAG3′<br><br>R- 5′ GGAAAGCAAGTGAACGACAAC3′ |
| 9 | HDH | haloacid dehalogenase-like hydrolase (LinJ.28.1480) | Hydrolase activity | F-5′TGACCAGAATGCCCATCAAG3′<br><br>R-5′GCTGGTGATAATGTAGTCCGG3′ |
| 10 | AUT | Autophagy-related protein (LinJ.19.0860) | Autophagy-related protein ATG8/AUT7/APG8/PAZ2, putative (ATG8B.3) | F-5′CCTCTCCAGACCACCAATGTCC3′<br><br>R-5′GGCGAGCGTCACCTTCTTGA3′ |
| 11 | gMDH | glycosomal malate dehydrogenase (LinJ.19.0710) | glycolysis, malate metabolic process | F-5′GCGTGGATGTGTTTGTGATGGT3′<br><br>R-5′TTCGTCACAATGCAGAACACCG3′ |
| 12 | ItRS | Isoleucyl-tRNA synthetase (LinJ.36.5870) | isoleucyl-tRNA aminoacylation, tRNA aminoacylation for protein translation, translation | F-5′CCGCTGGATCGACTTTGACAAC 3′<br><br>R-5′AGGAAGTATGTGTTGGGGTCGG3′ |
| 13 | AC | Adenylate cyclase-like protein | cyclic nucleotide biosynthetic process, intracellular signaling | F-5′CGGTATGGAAAGGTGTACGAAG3′ |

| | | | | |
|---|---|---|---|---|
| | | (LinJ.28.0090) | pathway, transport Purine metabolism | R-5′TCGATGTATGACTGCAAGACG3′ |
| 14 | CYPs | Cytochrome p450-like protein (LinJ.34.3110) | Electron carrier activity, heme binding, monooxiginase activity | F-5′TACATCCCCTTCAGCTGCGG3′<br><br>R-5′CGCTCGTAAACCTCGCCAAC3′ |
| 15 | CBS | cystathionine beta-synthase (LinJ.17.0300) | Endogenous control gene | F-5′GAAGTACACGGTGGAGGCTG3′<br>R-5′CGCTGATCACGACCTTCTTC3′ |
| 16 | GAPDH | Glyceraldehydes-3 Phosphatedehydrogenase (LinJ.30.2990) | Endogenous control gene | F-5′CGCCGATGTCAACTGGATG3′<br>R-5′GCTCCTTCTTCAGCGTGTCG3′ |

**Table S2: Pattern of up-regulated and down-regulated gene expression in K133 AS-R parasite:**

| Fold changes | AS-R, Up-regulated | AS-R, Down regulated |
|---|---|---|
| >2.0-3.0 | 70 | 85 |
| >3.0 – 4.0 | 20 | 16 |
| >4.0 | 12 | 5 |
| Total genes | 102 | 106 |
| Percent of modulated genes | 1.11% | 1.15% |

**Footnote:** The percent modulated genes calculated from the total 9,170 genes obtained in QC after filter

**Table S3: Genes differentially modulated with their functional categories in K133 AS-R *L. donovani* parasites.**

| S.NO | GENE ID | FOLD CHANGE K133AS-R/ K133WT | REGULA-TION | DESCRIPTION |
|---|---|---|---|---|
| **NUCLEIC ACID METABOLISM** | | | | |
| 1. | LmjF23.snRNA.01 | 5.49 | UP | small nuclear RNA, U1 snRNA |
| 2. | LinJ.32.2210 | 2.81 | UP | Hypothetical protein , conserved catalytic activity, hydrolase activity, zinc ion binding |
| 3. | LinJ.35.2650 | 2.49 | UP | Hypothetical protein , unknown function DNA binding |
| 4. | LmjF23.snRNA.02 | 2.36 | UP | small nuclear RNA, U3 snRNA |
| 5. | LinJ.06.0750 | 2.30 | UP | Hypothetical protein , conserved Regulation of transcription , DNA binding |
| 6. | LinJ.15.0140 | 2.27 | UP | hypothetical protein, conserved nucleic acid binding, zinc ion binding |
| 7. | LinJ.05.0830 | 2.12 | UP | Methylthioadenosinephosphorylase, putative transferase activity, transferring pentosyl groups |
| 8. | LinJ.33.0580 | 2.09 | UP | Hypothetical protein , conserved RNA ligase activity |
| 9. | LinJ.25.1350 | 2.03 | UP | DNA-directed RNA polymerase ii Transcription |
| 10. | LmjF31.snRNA.01 | 2.01 | UP | small nuclear RNA, U2 snRNA |
| 11. | LinJ.36.1680 | 3.79 | DOWN | Universal minicircle sequence binding protein, putative; nucleic acid binding, zinc ion binding |
| 12. | LmjF27.rRNA.25 | 3.76 | DOWN | 28S ribosomal RNA (LSU-beta) |
| 13. | LinJ.28.1000 | 3.30 | DOWN | Endonuclease/exonuclease/phosphatase-like protein; endonuclease &exonuclease activity |
| 14. | LinJ.36.2040 | 3.20 | DOWN | Nucleoside transporter 1, putative nucleoside transmembrane transporter activity; |

| | | | | |
|---|---|---|---|---|
| | | | | guanosine salvage; inosine salvage. |
| 15. | LinJ.33.3390 | 2.93 | DOWN | h1 histone-like protein |
| 16. | LinJ.29.0260 | 2.67 | DOWN | Thymine-7-hydroxylase, putative<br>Iron ion binding; oxidoreductase activity. |
| 17. | LinJ.36.1710 | 2.29 | DOWN | Poly-zinc finger protein 2, putative<br>Nucleic acid binding ; Zinc ion binding |
| 18. | LinJ.33.3340 | 2.22 | DOWN | Small nuclear ribonucleoprotein SmD2<br>RNA splicing |
| 19. | LinJ.36.0800 | 2.22 | DOWN | Uncharacterized protein<br>Metal ion binding |
| 20 | LinJ.30.3630 | 2.12 | DOWN | Zinc finger-domain protein, putative<br>nucleic acid binding ; zinc ion binding |
| **PROTEIN , AMINO ACID METABOLISM** | | | | |
| 21. | LinJ.33.0320 | 3.57 | UP | Autophagy-related protein 3<br>autophagy ; protein transport |
| 22. | LinJ.18.1130 | 2.95 | UP | Hypothetical protein, conserved<br>zinc ion binding |
| 23. | LinJ.32.3080 | 2.91 | UP | Tubulin-tyrosine ligase-like protein<br>tubulin-tyrosine ligase activity<br>protein modification process |
| 24. | LinJ.36.3230 | 2.72 | UP | Putative lipoate protein ligase<br>ligase activity ; octanoyltransferase activity ; cellular protein modification process ; lipoate biosynthetic process |
| 25. | LinJ.36.0590 | 2.66 | UP | Ubiquitin-like protein, putative<br>damaged DNA binding<br>nucleotide-excision repair, proteasomal ubiquitin-dependent protein catabolic process |
| 26. | LinJ.27.0310 | 2.45 | UP | Methylmalonyl-coenzyme a mutase, putative |
| 27. | LinJ.19.1560 | 2.34 | UP | Peptidylprolylisomerase-like protein<br>isomerase activity ; protein folding |
| 28. | LinJ.31.0290 | 2.16 | UP | Hypothetical protein,<br>L-ascorbic acid binding, iron ion binding, oxidoreductase activity |
| 29. | LinJ.33.1410 | 2.07 | UP | Cysteine conjugate beta-lyase,<br>aminotransferase- like protein |

| | | | | |
|---|---|---|---|---|
| | | | | lyase activity ; pyridoxal phosphate binding ; transaminase activity ; biosynthetic process |
| 30. | LinJ.04.0160 | 7.11 | DOWN | Cell adhesion ; metalloendopeptidase activity |
| 31. | LinJ.19.0860 | 5.32 | DOWN | Autophagy-related protein autophagy |
| 32. | LinJ.09.0180 | 4.28 | DOWN | Autophagy-related protein autophagy |
| 33. | LinJ.20.1320 | 3.86 | DOWN | calpain-like cysteine peptidase, putative,calpain-like cysteine peptidase, Clan CA, family C2 |
| 34. | LinJ.19.0820 | 3.83 | DOWN | Autophagy-related protein Autophagy |
| 35. | LinJ.20.1220 | 2.47 | DOWN | Putative calpain-like cysteine peptidase calcium-dependent cysteine-type endopeptidase activity ;proteolysis. |
| 36. | LinJ.20.1210 | 2.37 | DOWN | Putative calpain-like cysteine peptidase calcium-dependent cysteine-type endopeptidase activity ; proteolysis. |
| 37. | LinJ.11.0640 | 2.18 | DOWN | Putative aminopeptidase aminopeptidase activity ; manganese ion binding ; metalloexopeptidase activity ; proteolysis. |
| 38. | LinJ.36.6600 | 2.13 | DOWN | Ubiquitin-protein ligase, putative acid-amino acid ligase activity ; protein modification process ; ubiquitin-protein transferase activity |
| 39. | LinJ.33.2670 | 2.03 | DOWN | Putative carboxypeptidase metallocarboxypeptidase activity ;proteolysis. |
| 40. | LinJ.31.1140 | 2.01 | DOWN | Putative N-acyl-L-amino acid amidohydrolase metallopeptidase activity ;protein dimerization activity;proteolysis ;aminoacylase activity |
| **TRANSLATION** | | | | |
| 41. | LmjF21.0960 | 3.18 | UP | Hypothetical protein, conserved ATP binding ; aminoacyl-tRNA ligase activity |
| 42. | LmjF27.rRNA.32 | 8.27 | DOWN | 28S ribosomal RNA (LSU-delta, M2) |

| | | | | |
|---|---|---|---|---|
| 43. | LinJ.33.2880 | 2.54 | DOWN | Translation initiation factor IF-2, putative GTP binding ;translation initiation factor activity; GTPase activity. |
| 44. | LinJ.36.5870 | 2.43 | DOWN | Isoleucyl-tRNAsynthetase, putative ATP binding ;aminoacyl-tRNA ligase activity ; isoleucine-tRNA ligase activity ; nucleotide binding; zinc ion binding. |
| 45. | LinJ.36.1490 | 2.14 | DOWN | Translation elongation factor 1-beta, putative translation elongation factor activity |
| **LIPID METABOLISM** | | | | |
| 46. | LinJ.14.1450 | 2.59 | UP | Myo-inositol-1-phosphate synthase,inositol-3-phosphate synthase, putative (INO1) Inositol biosynthetic process, metabolic process, phospholipid biosynthetic process |
| 47. | LinJ.13.0200 | 2.25 | UP | Hypothetical protein, triglyceride lipase activity, zinc ion binding hydrolase activity ; lipid metabolic process . |
| 48. | LmjF19.1345 | 2.64 | DOWN | Glycerol uptake protein, putative integral component of membrane [GO:0016021] |
| 49. | LinJ.14.0670 | 2.13 | DOWN | Fatty acid elongase, putative fatty acid biosynthetic process, long-chain fatty acid biosynthetic process |
| 50. | LinJ.03.0280 | 2.13 | DOWN | Hypothetical protein, (pseudogene) fatty acid biosynthetic process ; iron ion binding; oxidoreductase activity |
| **CARBOHYDRATE METABOLISM** | | | | |
| 51. | LinJ.36.6550 | 3.48 | DOWN | Glucose transporter, lmgt2 fructose transmembrane transporter activity ; galactosetransmembrane transporter activity; glucose transmembrane transporter activity ; mannose transmembrane transporter activity ; glucose import |
| 52. | LinJ.22.0002 | 3.43 | DOWN | Hypothetical protein |
| 53. | LmjF06.1290 | 3.37 | DOWN | Hypothetical protein, heme binding, transition metal ion binding |

| | | | | |
|---|---|---|---|---|
| 54. | LinJ.34.4290 | 3.20 | DOWN | Lipophosphoglycan biosynthetic protein (lpg2)<br>GDP-mannose transmembrane transporter activity<br>nucleotide-sugar transport |
| 55. | LmjF36.6290 | 3.15 | DOWN | Glucose transporter, lmgt2<br>fructose transmembrane transporter activity ;<br>galactosetransmembrane transporter activity ;<br>glucose transmembrane transporter activity ;<br>mannose transmembrane transporter activity ;<br>carbohydrate transport |
| 56. | LinJ.36.6560 | 3.10 | DOWN | Glucose transporter, lmgt1<br>fructose transmembrane transporter activity ;<br>galactosetransmembrane transporter activity;<br>glucose transmembrane transporter activity;<br>mannose transmembrane transporter activity |
| 57. | LinJ.34.4360 | 2.38 | DOWN | D-isomer specific 2-hydroxyacid dehydrogenase- like protein |
| 58. | LmjF34.1400 | 2.33 | DOWN | D-isomer specific 2-hydroxyacid dehydrogenase- protein |
| 59. | LmjF07.0805 | 2.01 | DOWN | phosphoacetylglucosaminemutase-like protein,acetylglucosaminephosphomutase, putative,N-acetylglucosamine-phosphate mutase, putative |
| **CELL MOVEMENT** | | | | |
| 60. | LinJ.25.2050 | 2.96 | UP | Kinesin, putative<br>ATP binding, microtubule motor activity |
| 61. | LinJ.18.0530 | 2.15 | UP | Hypothetical protein, conserved<br>GTPase activator activity ; cell morphogenesis |
| 62. | LinJ.06.1070 | 2.12 | UP | Kinesin, putative<br>ATP binding, microtubule motor activity |
| 63. | LinJ.32.0240 | 2.93 | DOWN | Dynein light chain, flagellar outer arm, putative<br>microtubule-based process |
| 64. | LinJ.05.0290 | 2.62 | DOWN | ARP2/3 complex 16kDa subunit, putative<br>Arp2/3 complex-mediated actin nucleation ;<br>regulation of actin filament polymerization |
| 65. | LmjF05.0285 | 2.58 | DOWN | ARP2/3 complex 16kDa subunit, putative<br>regulation of actin filament polymerization |

| | | | | |
|---|---|---|---|---|
| 66. | LinJ.08.1280 | 2.38 | DOWN | Tubulin beta chain (Fragment)<br>GTP binding, GTPase activity, structural molecule activity ; microtubule-based movement ; microtubule-based process ; protein polymerization |
| 67. | LinJ.29.0520 | 2.17 | DOWN | Actin severing and dynamics regulatory protein<br>actin filament depolymerization |
| 68. | LinJ.23.1570 | 2.14 | DOWN | Putative dynein heavy chain<br>ATP binding, ATPase activity, microtubule motor activity |
| 69. | LinJ.05.0380 | 2.08 | DOWN | Putative microtubule-associated protein |
| 70. | LinJ.20.1340 | 2.04 | DOWN | Calpain-like cysteine peptidase, putative<br>small myristoylated protein-2, putative;(SMP-2)<br>flagellar cell motility ; flagellar pocket membrane. |
| 71. | LinJ.35.5310 | 2.04 | DOWN | Hypothetical protein, conserved |
| 72. | LinJ.33.2770 | 2.03 | DOWN | Dynein intermediate chain, putative |
| **CELL MEMBRANE PROTEIN** | | | | |
| 73. | LinJ.17.0020 | 5.56 | UP | integral component of membrane |
| 74. | LinJ.31.0460 | 5.36 | UP | Amastin, putative<br>integral component of membrane |
| 75. | LmjF31.0450 | 4.36 | UP | Amastin, putative |
| 76. | LinJ.31.1900 | 3.51 | UP | Hypothetical protein<br>integral component of membrane |
| 77. | LmjF08.0770 | 3.44 | UP | Amastin-like protein |
| 78. | LinJ.29.3030 | 3.37 | UP | Amastin, putative<br>integral component of membrane |
| 79. | LinJ.08.0690 | 3.35 | UP | Amastin-like protein<br>integral component of membrane |
| 80. | LmjF31.1855 | 3.35 | UP | Hypothetical protein |

| | | | | |
|---|---|---|---|---|
| | | | | integral component of membrane |
| 81. | LinJ.08.0680 | 3.30 | UP | Amastin-like protein<br>integral component of membrane |
| 82. | LinJ.08.0720 | 3.29 | UP | Amastin-like protein<br>integral component of membrane |
| 83. | LinJ.24.1300 | 3.18 | UP | Amastin-like surface protein-like protein<br>integral component of membrane |
| 84. | LinJ.08.0700 | 2.77 | UP | Amastin-like protein<br>integral component of membrane |
| 85. | LinJ.34.3430 | 2.62 | UP | Hypothetical protein (pseudogene)<br>integral component of membrane |
| 86. | LmjF34.0500 | 2.58 | UP | Amastin-like protein |
| 87. | LinJ.26.1440 | 2.54 | UP | Hypothetical protein, integral component of membrane |
| 88. | LinJ.34.1150 | 2.39 | UP | Amastin-like surface protein, putative<br>integral component of membrane |
| 89. | LinJ.29.3000 | 2.38 | UP | Amastin, putative<br>integral component of membrane |
| 90. | LinJ.26.2590 | 2.08 | UP | Hypothetical protein, conserved,<br>ATP binding; D-alanine-D-alanine ligase activity; metal ion binding; methyltransferase activity; peptidoglycan biosynthetic process |
| 91. | LinJ.30.2810 | 2.07 | UP | Hypothetical protein, conserved<br>integral component of membrane |
| 92. | LinJ.25.2390 | 2.05 | UP | Hypothetical protein, conserved<br>protein name -Palmitoyltransferase<br>protein-cysteine S-palmitoyltransferase activity ; zinc ion binding |
| 93. | LmjF31.1165 | 2.02 | UP | Hypothetical protein<br>integral component of membrane ; heme binding |
| 94. | LinJ.34.1690 | 2.01 | UP | Amastin-like surface protein, putative |
| 95. | LinJ.04.0170 | 4.68 | DOWN | Surface antigen-like protein |
| 96. | LinJ.21.1090 | 2.94 | DOWN | integral component of membrane |
| 97. | LinJ.05.0900 | 2.81 | DOWN | Surface antigen-like protein |

| | | | | |
|---|---|---|---|---|
| | | | | protein binding |
| 98. | LinJ.10.0070 | 2.52 | DOWN | Dehydrogenase-like protein integral component of membrane ;binding ; catalytic activity ; oxidoreductase activity. |
| 99. | LinJ.04.0180 | 2.37 | DOWN | Surface antigen-like protein |
| 100. | LinJ.04.0190 | 2.36 | DOWN | Surface antigen-like protein |
| 101. | LinJ.33.2980 | 2.34 | DOWN | integral component of membrane |
| 102. | LinJ.14.0500 | 2.26 | DOWN | integral component of membrane |
| 103. | LinJ.34.1050 | 2.13 | DOWN | Amastin-like protein |
| 104. | LinJ.33.0220 | 2.12 | DOWN | integral component of membrane |
| 105. | LinJ.08.0600 | 2.10 | DOWN | integral component of membrane |
| 106. | LinJ.24.1310 | 2.08 | DOWN | Amastin-like surface protein-like protein integral component of membrane |
| 107. | LinJ.23.1130 | 2.02 | DOWN | integral component of membrane |
| **CELL SIGNALLING** | | | | |
| 108. | LinJ.35.0780 | 3.36 | UP | CBS domain protein, conserved |
| 109. | LinJ.33.1930 | 3.15 | UP | Protein kinase, putative,dual-specificity protein kinase, putative ATP binding, protein kinase activity, protein serine/threonine kinase activity, protein tyrosine kinase activity |
| 110. | LinJ.22.1450 | 3.11 | UP | Ser/thr protein phosphatase, putative hydrolase activity |
| 111. | LinJ.25.1410 | 2.99 | UP | Hypothetical protein, conserved regulation of signal transduction |
| 112. | LinJ.17.0440 | 2.08 | UP | Protein kinase, putative ATP binding, protein kinase activity, protein serine/threonine kinase activity, protein |

| | | | | |
|---|---|---|---|---|
| | | | | tyrosine kinase activity |
| 113. | LinJ.34.0840 | 2.39 | DOWN | Serine/threonine-protein phosphatase metal ion binding; phosphoprotein phosphatase activity . |
| 114. | LinJ.16.0240 | 2.38 | DOWN | Protein tyrosine phosphatase-like protein protein tyrosine phosphatase activity, protein tyrosine/serine/threonine phosphatase activity protein dephosphorylation |
| 115. | LinJ.28.0090 | 2.26 | DOWN | Adenylatecyclase-like protein Adenylatecyclase activity ; heme binding ; oxygen binding ; intracellular signal transduction |
| **TRANSPORTER** | | | | |
| 116. | LinJ.31.0030 | 6.33 | UP | Aquaglyceroporin (AQP1) metal ion transmembrane transporter activity |
| 117. | LinJ.18.1270 | 2.70 | UP | Hypothetical protein, conserved binding; transport. |
| 118. | LinJ.11.0520 | 2.60 | UP | Nucleobase transporter (NT4) Nucleobase transmembrane transporter activity |
| 119. | LinJ.18.0040 | 2.54 | UP | Transporter, putative,major facilitator superfamily protein (MFS), putativetransporter activity |
| 120. | LinJ.18.0400 | 2.31 | UP | UDP-galactose transporter (LPG5B) sugar: hydrogensymporter activity; nucleotide-sugar transport |
| 121. | LmjF06.0080 | 2.27 | UP | ATP-binding cassette protein subfamily G, member 1, putative (ABCG1), ATP binding, ATPase activity, coupled to transmembrane movement of substances, phosphopantetheine binding |
| 122. | LinJ.10.0900 | 2.17 | DOWN | Nuclear transport factor 2, putative,ntf2-like transport |
| 123. | LinJ.33.3410A | 2.16 | DOWN | ABC transporter family-like protein ATPase activity ; ATP binding |
| 124. | LinJ.07.1340 | 2.15 | DOWN | Amino acid transporter, putative (AAT19) |

| | | | | |
|---|---|---|---|---|
| 125. | LinJ.22.0100 | 2.11 | DOWN | Amino acid permease, putative (AAT22) |
| | | **STRESS PROTEIN** | | |
| 126. | LinJ.22.0670 | 5.13 | UP | A2 protein<br>protein binding ;cellular response to stress. |
| 127. | LinJ.18.1660 | 4.17 | UP | Gamma-glutamylcysteinesynthetase (GSH1)<br>glutathione biosynthetic process, response to reactive oxygen species, trypanothione biosynthetic process<br>glutamate-cysteine ligase activity |
| 128. | LinJ.31.0530 | 2.38 | UP | Hypothetical protein, conserved<br>heat shock protein binding ; unfolded protein binding;<br>protein folding . |
| 129. | LinJ.33.0940 | 2.25 | UP | dnaj chaperone-like protein<br>heat shock protein binding ;unfolded protein binding;<br>protein folding. |
| 130. | LinJ.28.1480 | 2.06 | UP | Haloaciddehalogenase-like hydrolase, putative<br>catalytic activity, hydrolase activity |
| | | **OXIDATION - REDUCTION** | | |
| 131. | LinJ.31.1190 | 2.56 | UP | Hypothetical protein, heme binding ;<br>transition metal ion binding |
| 132. | LinJ.25.2150 | 2.40 | UP | Hypothetical protein, conserved<br>electron carrier activity, oxidoreductase activity<br>2 iron, 2 sulfur cluster binding; nitrite reductase [NAD(P)H] activity ; nitrate assimilation |
| 133. | LinJ.34.3110 | 2.19 | DOWN | Cytochrome p450-like protein<br>electron carrier activity ; heme binding; monooxygenase activity |
| 134. | LinJ.19.1490 | 2.14 | DOWN | oxidoreductase-like protein<br>electron carrier activity, oxidoreductase activity |
| | | **OTHERS** | | |

| | | | | |
|---|---|---|---|---|
| 135. | LinJ.21.0070 | 5.64 | UP | Hypothetical protein, conserved |
| 136. | LinJ.18.0610 | 3.20 | UP | Hypothetical protein, conserved<br>zinc ion binding |
| 137. | LinJ.28.1700 | 2.52 | UP | Hydrolase, alpha/beta fold family, putative<br>hydrolase activity |
| 138. | LinJ.33.0750 | 2.46 | UP | Hypothetical protein, conserved |
| 139. | LinJ.29.3020 | 2.02 | UP | Tuzin like protein, putative |
| 140 | LinJ.06.1350 | 2.98 | DOWN | Hypothetical protein<br>heme binding; transition metal ion binding. |
| 141. | LinJ.01.0010 | 2.57 | DOWN | Hypothetical protein<br>binding |
| 142. | LinJ.36.4270 | 2.33 | DOWN | Phosphoglyceratemutase family member 5, putative<br>protein serine/threonine phosphatase activity. |
| 143. | LinJ.02.0280 | 2.24 | DOWN | Hypothetical protein, conserved<br>binding |
| 144. | LinJ.33.2460 | 2.07 | DOWN | Hypothetical protein,<br>catalytic activity; metabolic process. |

**Figure S2.**

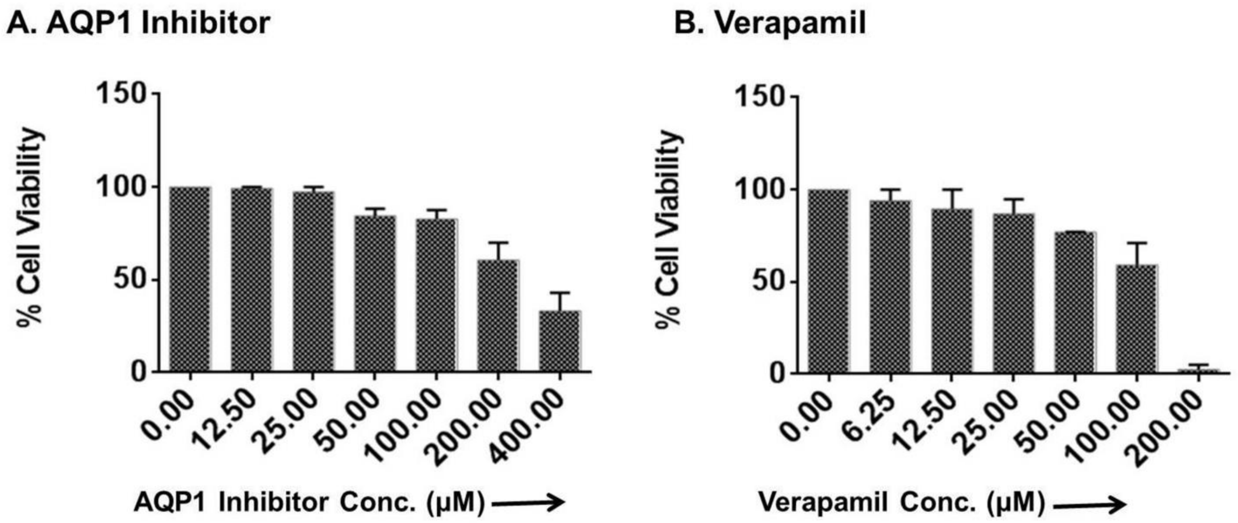


Figure S2: **Cytotoxicity of (A) AQP1 inhibitor and (B) Verapamil to host macrophages (mice PECs).** % cell viability ± SD with the increasing drug concentration has been plotted here. The data was obtained from three independent experiments.
